# Mechanisms of immune evasion by head and neck cancer stem cells

**DOI:** 10.3389/froh.2022.957310

**Published:** 2022-08-02

**Authors:** Flávia Caló Aquino Xavier, Jamerson Carvalho Silva, Camila Oliveira Rodini, Maria Fernanda Setubal Destro Rodrigues

**Affiliations:** ^1^Laboratory of Oral Surgical Pathology, School of Dentistry, Federal University of Bahia, Salvador, BA, Brazil; ^2^Department of Biological Sciences, Bauru School of Dentistry, University of São Paulo, São Paulo, SP, Brazil; ^3^Postgraduate Program in Biophotonics Applied to Health Sciences, Nove de Julho University (UNINOVE), São Paulo, SP, Brazil

**Keywords:** head and neck squamous cell carcinoma (HNSCC), cancer stem cell (CSC), immune surveillance, immune evasion, immunotherapy

## Abstract

Different mechanisms are involved in immune escape surveillance driven by Oral and Head and Neck Cancer Stem Cells (HNCSCs). The purpose of this review is to show the most current knowledge regarding the main impact of HNCSCs on tumor evasion through immunosuppression, CSCs phenotypes and environmental signals, highlighting strategies to overcome immune evasion. The main results drive the participation of cell surface receptors and secreted products and ligands, the crosstalk between cells, and genetic regulation. The reduction in CD8^+^ T cell recruitment and decreased effector of anti-PD-1 therapy by cells expressing BMI1 is a key event; Natural Killer cell ligands and cytokines needed for its activation and expansion are crucial to control tumor growth and to target CSCs by immunotherapy; CSCs expressing ALDH1 are related to increased expression of PD-L1, with a positive link between DNMT3b expression; CD276 expression in CSCs can act as a checkpoint inhibitor and together with Activator Protein 1 (AP-1) activation, they create continuous positive feedback that enables immune evasion by suppressing CD8+ T cells and prevent immune cell infiltration in head and neck cancer. These data demonstrate the relevance of the better understanding of the interaction between HNCSCs and immune cells in the tumor microenvironment. The ultimate clinical implication is to ground the choice of optimized targets and improve immune recognition for ongoing treatments as well as the response to approved immunotherapies.

## Introduction

Cancer cells generally collapse the development of a specific antitumor immunity as a survival strategy, leading to “immune evasion mechanisms” that guarantee the success of tumor formation and progression [1]. Immune effector cells have cytotoxicity repressed in the tumor microenvironment due to different mechanisms driven by distinct cellular interactions and secreted factors [2]. In this context, recent studies have demonstrated that Cancer Stem Cells (CSCs) represent immune-privileged cells able to initiate tumor growth and mediate metastasis, tumor recurrence, and therapeutic resistance [3, 4].

CSCs are a non-immunogenic long-lived cell type that represents a relevant target cell population for mutations to occur until the development of an immune escape phenotype [5]. These cells survive all phases of the immune editing process, enabling them to efficiently modulate immune responses and avoid immune-mediated destruction [5]. CSCs can present a deficient expression of human leukocyte antigens (HLA)- A, B, and C and also antigen-processing machinery (APM) molecules in different types of cancer, which implicates in non-recognition by CD8^+^ T cells [4]. CSCs can avoid Natural Killer (NK) cytotoxicity by the low expression of ligands necessary for their activated state, like natural killer group 2D (NKG2D) ligands [4]. Therefore, the immune system defenses cannot defeat CSCs in the elimination phase. Consequently, these cells remain fully protected, achieving a dynamic balance between their quiescent state and augmented resistance to cell killing [6]. In this phase, CSCs acquire genetic and epigenetic alterations but are still niche confined and constrained by immune cells, which prevent the establishment of clinically relevant tumors [7]. However, less immunogenic and immunosuppressive CSCs clones emerge and expand in the equilibrium phase. When the immune system function is impaired by age, therapy, or disease, it uses this opportunity to divide itself. Asymmetric cellular division occurs during this process, and the recruitment of immunosuppressive cells to the tumor microenvironment (TME) favors rapid tumor growth [4].

In the TME, CSCs interact with different cell types to favor its immune evasion [8]. Dendritic cell (DC) recruitment, maturation, and differentiation are impaired by CSCs mainly *via* increased secretion of TGF-β, which leads to the downregulation of CD80, CD86, and MHC class II molecules in DC, which are responsible for the co-stimulatory activity, as well as the development of PD-L^+^ DC that contributes to immunotherapy resistance [9]. CSCs positively correlate with T regulatory lymphocytes (Treg), a population of CD4^+^ T cells that contribute to tumor stemness and progression mainly *via* inhibiting effector T cells and secretion of IL-4, IL-10, IL-35, and TGF-β, cytokines types with an immunosuppressive function [10, 11]. Moreover, the recruitment of myeloid-derived suppressor cells (MDSC) and tumor-associated macrophages (TAM) into the TME by CSCs and their constant interaction, contribute not only to the establishment of an immunosuppressive TME and increased expression of PD-1 and PD-L1 by T lymphocytes and CSCs, respectively, but also promotes CSCs maintenance and survival *via* different mechanisms, including activation of mTOR, NF-κB, STAT3, and Src signaling pathways and secretion of different cytokines [12–14]. It is also essential to highlight that HLA-I or low expression of the APM by CSCs is another relevant mechanism by which they are poor targets for T cell-mediated immune response [15].

Head and neck squamous cell carcinoma (HNSCC) is the sixth-ranked worldwide most common cancer, characterized by very aggressive behavior and poor prognosis [16, 17]. Conventional treatment is associated with morbidity, toxicity, and discrete improvement in overall survival [18]. HNSCC is a heterogeneous group of malignancies with their origin from different anatomic subsites. In addition, they present a diversity of risk factors and a broad molecular profile, imposing difficulties in the study and treatment of these tumors [17]. HNSCC shows, among other solid tumors, one of the most inflamed TME and has a high tumor mutation burden, which may benefit from immunotherapy strategies [19]. Recently, the use of nivolumab and pembrolizumab (anti-PD-1 immune checkpoint inhibitors) in patients with recurrent HNSCC has demonstrated improved outcomes compared to standard therapy [20].

However, single-agent strategies in immunotherapy have caused either temporary or lasting responses only in a minor subset of HNSCC patients [21]. Mapping how head and neck cancers overcome immune surveillance within TME provides optimal strategies to better deal with this tolerance [21]. Therefore, understanding the cross-talk between HNCSC and the immune system is extremely important as these cells directly impact tumor development, progression, and response to therapy. Thus, this mini-review aims to present how HNCSCs contribute to immune evasion leading to immunosuppression and to the emergence of genetic and epigenetic genotypes with immune privilege and points out some strategies to overcome immune evasion.

## HNCSCs and escape from the host immune surveillance

CSCs maintain a cross-talk with immune cells in the TME to promote an immunosuppressive milieu that allows tumor development as a result of escape from the host's immune surveillance [22]. However, the mechanisms displayed by CSCs that enable their survival under immune vigilance during HNSCC tumorigenesis and metastasis are not well-established [23]. Recent studies demonstrate a dual function of CSCs in the immune system. First, the outgrowth of these cells can elicit immune system responses to destroy them. Instead, immunoediting generates CSCs to survive even in immunocompetent patients or provide necessary conditions within the TME, allowing tumor progression [24].

**Figure 1:**
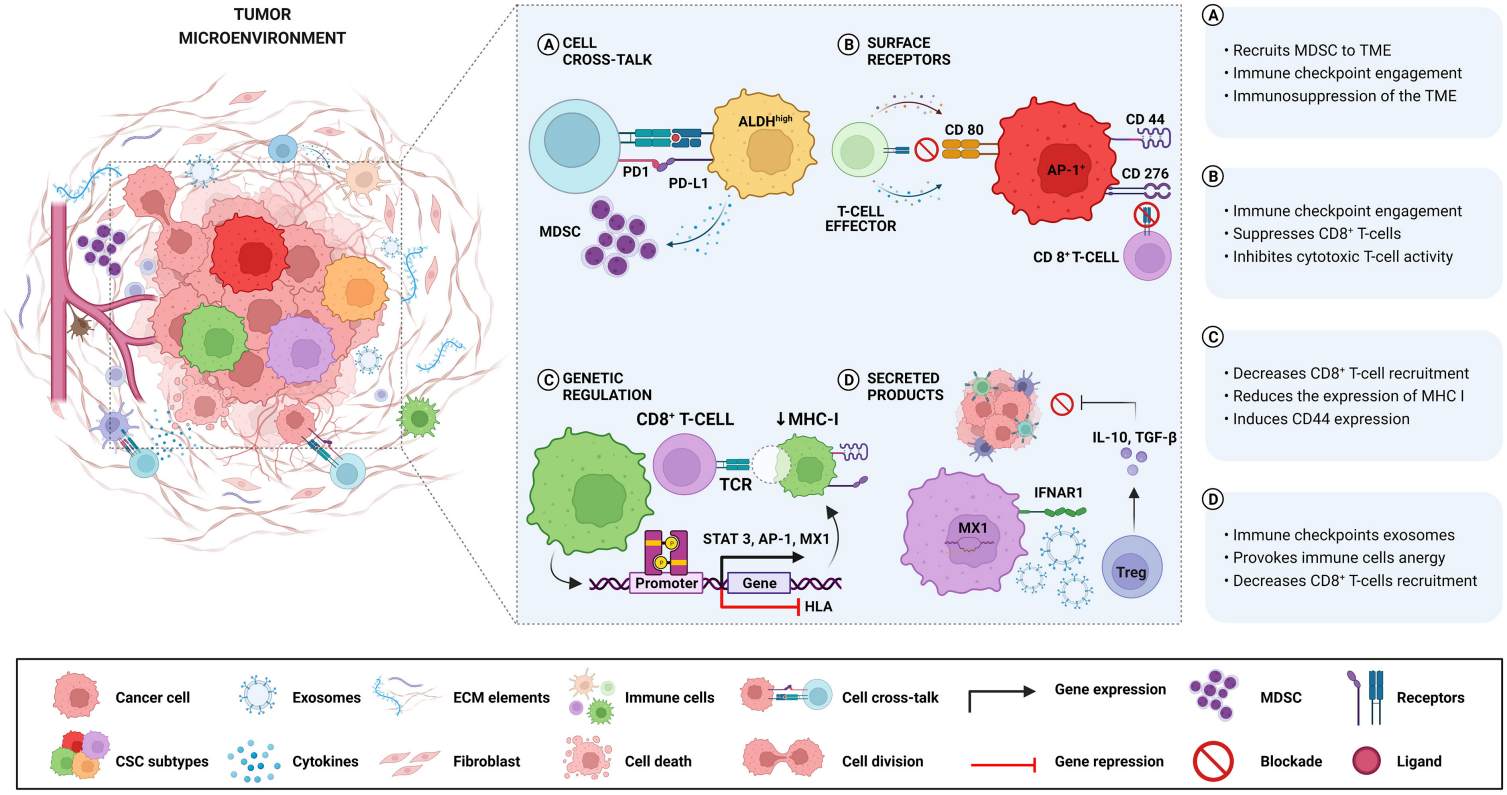
Different mechanisms involved in immune escape are driven by Oral and HNCSCs. **(A)** ALDH^high^-CSCs exhibit higher levels of PD-L1 and recruit MDSC with a suppressive role, causing negative regulation of immune responses in the TME. **(B)** CSCs expressing CD276 and AP-1 create a continuous positive feedback that enables immune evasion by suppressing CD8^+^ T cells. After TGF-β stimulation, CSCs express CD80 and inhibit T cell cytotoxicity leading to resistance to T cell immunotherapy. **(C)** The activation of the STAT3 pathway is related to the expression of PD-L1 in CD44^+^ cells, resulting in immune escape played by CSCs. Downregulation of HLA genes in CSCs decreases the expression of MHC class I causing non-recognition by T cells. **(D)** High expression of MX1 in CSCs decreases CD8+ T-cells infiltration concomitant with IFNAR1 expression associated with the release of exosomes containing immune checkpoint receptors. Noteworthy, the same CSCs can activate more than one immune avoidance mechanism.

Different mechanisms involved in immune escape driven by Oral and HNCSCs are summarized in Table 1 and Figure 1. Wang et al. [23] demonstrated that expression of CD276 and Activator Protein 1 (AP-1) created continuous positive feedback to enable immune evasion, self-renewal, and metastasis by CSCs in HNSCC. Miao et al. [35] reported that CSCs express CD80 to interact with T lymphocytes after Transforming Growth Factor-beta (TGF-β) impulse. They also showed that CD80 surface ligand on CSCs directly inhibited T cells cytotoxicity and mediated resistance to approaches with immunotherapies. Gong et al. [33] revealed that worse survival was related to a higher expression of MX1 (MX dynamin-like GTPase 1) and relied on the amount of CD8^+^ T-cells in HNSCC, including oral cavity cancers. In the same study, a cancer-specific IFN-I receptor (IFNAR1) provided a stemness state and the release of exosomes derived from CSCs carrying receptor ligands associated with immune checkpoint function.

**Table 1:** The main immune evasion mechanisms and strategies to overcome immune evasion in oral and head and neck cancer stem cells.

**References**	**Article type**	**Stem cell marker**	**Immune evasion mechanism**	**Strategy to overcome immune evasion**	**Main findings**	**Origin of cells/tumor samples**
Tseng et al. [25]	Experimental study	Augmented expression of CD44 and CD133 plus downregulated expression of PD-L1 and EGF-R	Crosstalk among CSCs, monocytes and NK cells reduces immune response; saving CSC of NK cells lyse process, in a manner dependent on several cytokines combination.	It was suggested that repeated allogeneic NK cell transplantation may eliminate cancer stem cells and overcome the patient NK cells modified phenotype induced by CSC	NK cell activity may be crucial to induce tumor cells to a more differentiated state by secreting critical cytokines, making the tumor cells more targetable to current treatments	Patient-derived primary oral squamous cancer stem cells from freshly resected tongue tumors (UCLA-OSCSCs)
Visus et al. [26]	Experimental study	ALDH1/3 isoforms	HLA class I Ag lack of expression in CSC is associated with reduced CD8+ cell function	ALDH1A1-specific CD8+ T-based immunotherapy to selectively target CSC	CD8+ T cells targeted to ALDH1A1 positive cells caused their elimination and prevent tumor growth and dissemination plus an increased the rate of survival.	Human SCCHN cell line was established at the University of Pittsburgh Cancer Institute
Jewett et al. [27]	Review	CD133 and CD44	CSC has a suppressive influence on NK cell activity; the Fas ligand is one of the responsible for tumor-associated NK cells' decreased cytotoxicity. In addition to this, loss of mRNA for granzyme B and lack of CD16 and its associated zeta chain plus NF-κB activation also contribute.	Targeting NF-κB, which seems to be related to cancer progression, to improve NK cell-mediated cytotoxicity against oral tumors. This could be achieved by continuous infiltration of allogeneic NK cells to target CSC	CSC can persuade NK cells to release cytokines to benefit tumor progression and spread, associated with more accuracy to detect differentiated cells displayed by NK cells instead of CSC. Thus, different strategies can overcome this hindrance, one targeting CSC and the other dictated to more differentiated cancer cells.	Cell lines and human samples of HNSCC
Qian X et al. [24]	Review	ALHD1, CD44	Lacking expression of cell surface MHC 1 by CSC, which reduces immune response by CD8+ lymphocyte	Vaccines containing lysates of CSCs-enriched tumor cells or CD8+ activated lymphocytes against CSC's antigens	CSC recognized by the host immune system may evade immune surveillance and induce suppression. Ways to improve immune responses against CSC are explored for immunotherapy targeting specific antigens in these cells, as ALDH	Mainly CSCs from HNSCC
Lee et al. [28]	Experimental study	CD44	Expression of PD-L1 is induced by the binding of STAT3 on its gene promoter, which is constitutively phosphorylated on CD44-positive cancer cells	CD44+ cells have their PD-1 status reduced by STAT3 blockade. Anti-PD-1 therapy efficacy was recovered against once non-immunogenic CD44 cells	Sustained phosphorylation of STAT3 was related to a PD-L1 expression on CD44+ cells, enabling these cells to bypass immune surveillance, and providing mechanisms to maintain tumor quiescence making possible relapse after treatment	Human samples from HPV-negative oral cavity SCC
Prince et al. [29]	Experimental study	ALDH	CSC that shows HLA-ABC downregulated molecules represents one of the ways by which immune vigilance can be misled in HNSCC	Stimulation of dendritic cells with CSC lysate preparations to generate a specific immune response to CSC	It is an applicable option to use dendritic cells stimulated with CSC lysate from HNSCC to prepare ALDH^high^-DC (CSC-DC) as an anti-CSC therapy.	Established cell line (HUM00042189) from patients with HNSCC enrolled in the University of Michigan SPORE and HNSCC cell line assigned as UMHNSCC-237
Tsai et al. [30]	Experimental study	ALDH1	CSC expressing ALDH1 was related to expression PD-L1 and recruitment of MDSC, with a positive link between ALDH1 and DNMT3b expression	DNA hypomethylating agents as epigenetic therapy decreased ALDH1 expression and induces DNA damage. In addition, MDSCs and the expression of PD-L1 were significantly attenuated	ALDH1 may function by epigenetic mechanisms, which can be targeted by epigenetic therapy approaches.	Human samples derived from OSCC (stage III-IV)
Kaur et al. [31]	Experimental study	Augmented expression of CD44 and CD133 plus downregulated expression of PD-L1 and EGF-R	CSC expressed lower levels of MHC class 1, NK-activating ligands associated with the deficient release of crucial cytokines with NK cell expansion action	Stimulation of NK cells with osteoclasts induces CSC lysis by expanding NK cells and increasing their cytotoxicity and IFN-γ secretion, thus, forcing CSC to express MHC I and enhance their interactions with CD8+ T cells	NK cells are boosted more efficiently by osteoclasts. NK cell cancer-patient-derived are less reactive when compare to healthy donators. Others strategies to expand NK cells do not show better results rather when OCs are used as feeders to these immune cells. Efficient control of tumor growth can be established with this novel protocol.	Tissue samples from cancer patients with tongue tumors.
Sanmamed and Chen [1]	Review	PD1	PD1 signaling inhibits lymphocyte T cytotoxicity when present within the tumor	Anti-PD-1 antibodies combined with other therapeutic approaches	Restoring immune response against tumors by combined therapeutic approaches could improve and repair a once lost natural antitumor immune capacity.	Cell lines and human samples of HNSCC.
Jia et al. [32]	Experimental study	BMI1, SOX2, CD 80	Cells BMI1+ deceive CD8 T lymphocytes response plus relapse anti-PD-1 blockade. The CSC BMI 1 positive cells also repress the transcription of chemokines by chromatin repression with H2AUb in their promotors, leading to inhibition of CD8+ T cells recruitment.	BMI1 inhibitor associated with anti-PD1 therapy eliminates BMI1+ CSCs, and also improves CD8 lymphocytes T recruitment and secretion of IFN1, by removal of their repressive marker H2Aub on promoters	BMI1 specific inhibition pharmacologically or genetically was capable of eliminating CSC BMI1 + and leads to cellular immune activation against the tumor in addition to improvement of anti-PD-1 therapy, achieving inhibition of tumor growth, spread, and relapse.	Cell lines SCC1, SCC9, SCC22B, SCC23, HN13, SCC1R e SCC23R, and human HNSCC samples.
Gong et al. [33]	Experimental study	CD44 and ALDH	When in intrinsic activation of IFNAR1, cancer cells demonstrated a stemness state with a higher release of exosomes containing suppressive immune checkpoint receptor ligands, including PD-1, and fosters immune evasion	CSC ALDH and CD44 positive amount was bit by IFNAR1 deficit	A poor clinical outcome was observed when cancer cells exhibited IFNAR1 signaling. Tumor progression reduction was gained after the blockade of IFNAR1, which was accompanied by the recruitment of T cells and reduction of MDSCs infiltration.	Samples from patients with HNSCC of the larynx, oral cavity, oropharynx, and hypopharynx/other
Wang et al. [23]	Experimental study	CD267	CSC expressing CD276 might use it as an immune checkpoint to reduce specific cellular responses in HNSCC.	Anti-CD276 antibodies eliminated CSC and also enhance CD8 T cells activation, reducing tumor growth and metastasis	The checkpoint molecule CD276 expressed in CSC allows these cells to escape immune vigilance through tumor initiation, progression, and metastasis. Anti-CD276 therapy was able to inhibit tumor growth and metastasis, improving antitumor immunity	Human HNSCC cell lines and tissue samples
Jia et al. [34]	Experimental study	circFAT1	STAT3 activation induces upregulation of circFAT1, positively associated with cancer stemness and immune evasion	circFAT1 knockdown enhances the anti-PD1 effect by promoting CD8+ cell infiltration into the tumor microenvironment	The lack of CD8+ T cells in the tumor site after treatment can in part explain relapse to anti-PD1 therapy. circFAT1 can promote an immunosuppressive TME in HNSCC, and its block enhances immune therapies target PD-1 also improving CD8+ cells infiltration.	HNSCC samples derived from tongue cancer

*CD, cluster of differentiation; PD-L1, programmed death-ligand 1; EGF-R, epidermal growth factor receptor; CSC, cancer stem cells; NK, natural killer cell; UCLA-OSCSC, oral squamous carcinoma stem cells from University of California, Los Angeles; ALDH, aldehyde dehydrogenase; HLA, human leucocyte antigen; SCCHN, squamous cell carcinoma of the head and neck; NF-κB, nuclear factor kappa B; HNSCC, head and neck squamous cell carcinoma; MHC, major histocompatibility complex; STAT, signal transducer and activator of transcription; HPV, human papillomavirus; SCC, squamous cell carcinoma; HLA, human leukocyte antigen; CSC-DC, cancer stem cells stimulated dendritic cells; SPORE, Special Project of Research Excellence; MDSC, myeloid-derived suppressor cells; DNMT3b, DNA methyltransferase 3 beta; OSCC, oral squamous cell carcinoma; IFN-γ, interferon gamma; OC, osteoclasts; BMI, B-cell-specific moloney murine leukemia virus integration; SOX2, SRY-box transcription factor 2; H2AUb, histone H2A monoubiquitylation; IFNAR1 interferon alpha and beta receptor subunit 1; circFAT1, circular RNA FAT1*.

A stemness profile has been linked to immune evasion. Cells with a CSCs phenotype undergoing EMT (Epithelial-Mesenchymal Transition) were correlated to ALDH1 activity [36]. Compared to ALDH negative cells, their positive counterparts demonstrated higher levels of PD-L1, with an increase after radiation treatment in OSCC [30]. Tsai et al. [30]. also evaluated the correlation between ALDH1 and MDSCs in OSCC. They reported that higher levels of PD-L1 were associated with tumors with ALDH1 positive expression in a combination of elevated levels of MDSCs. CD33^+^CD14^+^CD11b^+^HLA-DR^−^ cells had their percentage increased in ALDH1+ tumors. Furthermore, epigenetic therapy by injecting DNA methyltransferase (DNMT) inhibitor 5-aza-2′-deoxycytidine (5-AZDC) attenuated the radiation-induced PD-L1 expression in tumors [30]. The authors proposed that MDSC recruitment and high PD-L1 in ALDH1^+^ tumors may be responsible for resistance to radiotherapy. Additionally, this could be reversed by agents with DNA hypomethylation effect as it knockdown CSC properties and radioresistance [30].

Furthermore, Prince et al. [29] used peripheral blood mononuclear cells (PBMCs) from HNSCC patients and demonstrated unique CSCs antigens in the ALDH^high^ population cells isolated from the tumor specimen. The same research showed that DCs derived from PBMC and cultured in a preparation containing HNSCC ALDH^high^ cells could elicit responses in autologous B and T lymphocytes. ALDH^high^ CSCs induced antibodies and cytokine release and CTL activity. Hence, this strategy can guide CSC-DC vaccine production. Additionally, both humoral and cellular immunity against HNCSC was achievable, implying its potential for the treatment of HNSCC patients [29]. These findings were previously supported by Visus et al. [26] and indicate that CD8^+^ cells sensitized against ALDH1 positive HNCSC cells were able to target these cells and may contribute to tumor control. In fact, ALDH1A1-specific CD8^+^ T cells lysed ALDH^+^ cells, inhibiting tumor progression, and metastasis, and increasing the rate of survival of xenograft-bearing immunodeficient mice [26].

CD44 is another well-characterized marker associated with increased tumorigenesis, radioresistance, chemoresistance, and an immunosuppressive phenotype [28]. Lee et al. [28] showed that CD44^+^ cells were more immunosuppressive than their negative counterparts. This immunosuppression was partially switched when antibodies blocked the PD-1 receptor, suggesting a biologically and clinically relevant implication in PD-L1 expression between CD44^+^ and CD44^−^ cells. A protein-coding gene known as STAT3 sustains a phosphorylated state in CD44^+^ cells and its blockade decreases the expression of PD-L1. Therefore, the STAT3 pathway may be related to the expression of PD-L1 in CD44^+^ cells, resulting in an immune escape played by CSCs [28].

Moreover, as CSCs decrease the expression of MHC class I to evade T cell recognition, they become susceptible to Natural Killer (NK) cells [37]. Thus, based on NK cells' capacity to target CSCs, Kaur et al. [31] proposed a new protocol describing a sustainable and durable expansion of NK cells stimulated by osteoclasts with cytotoxicity activity against oral squamous carcinoma stem cells (OSCSCs). Besides eliminating CSCs, the infusion with super-charged NK cells control tumor growth and induces stem-like differentiation in poorly differentiated tumors targeted by immunotherapy. Furthermore, an expansion of T cells was noted when co-cultured with DCs. In contrast, osteoclasts expand NK cells, suggesting that NK and T cells respond to different stimuli in the TME. These findings have a translational focus, facilitating future cancer immunotherapies. NK cells derived from HNSCC patients exhibited a distinct profile when compared to healthy patients, with lower cellular lyse ability and reduced secretion of cytokines. Moreover, ligands and crucial cytokines for NK activation and expansion were lower in OSCSCs [31]. Previously, Tseng et al. [25] demonstrated that NK cell-mediated cytotoxicity quickly targets OSCSCs compared to their differentiated counterpart. Co-cultures of CSCs with NK cells demonstrated increased IFN-γ and low levels of GM-CSF and interleukins (IL-6 and IL-8). Most importantly, OSCSCs expressed CD133 and CD44^bright^ markers [25].

PD-L1 has shown a variable expression across patients with HNSCC, although success occurs in a subset of patients treated with therapies based on checkpoint inhibitors [38]. Wang et al. [23] demonstrated that the host immune vigilance is overcome when CSCs express other ligands related to the immune checkpoint, such as CD276 (B7-H3). CD276, as a stem cell marker, was suitable to isolate CSCs once it was expressed over this subpopulation of cells from mouse and human HNSCC. CD8^+^ lymphocytes recovered the cytotoxic potential against CSCs when anti-CD276 antibodies were infiltrated, hindering the spread of cells to lymph nodes and the tumor progression in animal models of HNSCC. Using Next-Generation Sequencing (NGS) techniques to better characterize this mechanism, the results showed that CD276 is crucial for CSCs immune evasion, and blockade with anti-CD276 eliminates these cells in a CD8^+^ T cell-dependent manner, in addition to remodeling HNSCC heterogeneity and decreasing EMT [23].

## Discussion

Even lymph nodes where high-density immune cells exist serve as niches for CSCs metastasizing in HNSCC [39]. This can be partly explained by the low immune response and augmented capacity to induce immunosuppression due to immunoediting imposed on tumor cells by effectors of the immune system [4]. Understanding the mechanisms involved in the interaction of Oral and HNCSCs and the immune system is extremely important and has been one of the most challenging questions in the new era of Immunology. These cells directly affect tumor development, progression, and response to therapy. The evolution of efficacious immunotherapies for head and neck cancer is based upon a deep knowledge of antitumor immunity and how these tumors evade immune surveillance. Despite the diversity of immune cells present in the tumor stroma, HNSSC developed many mechanisms by which antitumor immunity can be thwarted [21].

Tumoral heterogeneity has been supported by the presence and the maintenance of cancer cells with stemness ability. The CSC model proposed in many studies suggests that this specific subpopulation of cells within the tumor is responsible for disease progression and relapse after standard treatments [22]. The tumor progression requires different abilities by the subclones, which are present in cells that share stemness profile in Oral and HNSCC, giving a particular marker signature diversity that could result from its functional and molecular plasticity [40]. BMI1 is a crucial marker abnormally expressed in cells from HNSCC that shows self-renewal capacity [41]. The absence of the normal immune response on TME is majorly due to the lack of activated CD8^+^T cells. As previously reported, cytotoxic T cell activity is defeated by CSCs CD80^+^ and even resists adoptive cytotoxic T cell transfer (ACT)-based immunotherapy [35]. Wang et al. [23] also demonstrated that CD80 was expressed in BMI1+ CSCs, with decreased cytotoxic effect in lymphocytes. Additionally, Gong et al. [33] reported that CSCs signaling pathways simultaneously occur with an intrinsic activation of Interferon alpha and beta receptor subunit 1(IFNAR1) signaling, affecting the anti-tumor stimulation function of stimulator of interferon response cGAMP interactor 1 (STING1) on CD8+ lymphocytes by induction of a hypo-responsiveness status in HNSCC. Therefore, for properly targeting CSCs, detecting a panel of tumor-associated antigens specific to CSCs will amplify the potential of combinational therapies to target these cells effectively in HNSCC [24].

Furthermore, NK cells can significantly lyse CSCs, as shown by Jewett et al. [27]. When co-cultured with OSCSCs, an increase in NK cell cytotoxicity was noted compared with oral squamous carcinoma cells (OSCCs) in a more differentiated state. In addition, the tumor progression is allowed by the absence of effective responses of NK cells and the deficient stimulus for the expansion of CD8 lymphocytes [31]. Thus, continuous infusion of allogeneic NK cells in the TME may benefit patients with OSCC and is essential to controlling tumor growth [27, 31]. Noteworthy, IFN-γ secreted by NK cells induces differentiation of the CSCs, inducing the expression of MHC class I, essential to CD8^+^ T cell target function [31].

Little is known about whether immunotherapy against immune checkpoints could target CSCs [23]. Studies of PD-L1 (also known as CD274) expression in CSCs have yielded contradictory results in HNSCC [42], indicating that CSCs targeted with anti-PD/PD-L1 antibodies may not be as affected as thought, and other immune checkpoints can be involved. Wang et al. [23] recently showed that CSCs expressing CD276 in HNSCC orchestrate immune vigilance toward tumor initiation, progression, and metastasis. CD276 hindrance CD8^+^ T cells anti-tumor effects were improved with the elimination of CSCs by anti-CD276 immunotherapy, thus inhibiting tumor growth and spread. Importantly, CD276 blockade significantly inhibited lymph node metastasis of HNSCC, enhancing anti-tumor immunity [23]. Although CD276 was found to be upregulated in HNSCC, the molecular mechanism controlling CD276 expression remains unclear [23].

Targeting CSCs markers in HNSCC by PD1 blockade immunotherapy promotes CD8^+^ cell infiltration and improves cisplatin response [23, 34]. The anti-PD therapies applied to patients with HNSCC depend on the activated pathway, but in some cases, immune-related adverse events can occur, leading to treatment failure [1]. Since CSCs cause some of these events, identifying specific defects in the antitumor immune response and combining different target therapy approaches may improve treatment [1, 32, 34]. In this context, it is important to better characterize the CSCs markers in HNSCC to define optimized targets and improve ongoing treatments, especially OSCC immunotherapy. Following this idea, Tsai et al. [30] analyzed the link between ALDH1, PD-L1, and circulating MDSCs by FACS. These authors revealed ALDH1-positive tumors with high levels of circulating MDSCs, significantly incremented after radiotherapy. Furthermore, ALDH1-expressing tumor cells had higher PD-L1 expression, which was enhanced by radiation [30]. Wang et al. [23] also demonstrated in HNSCC an association between BMI1^+^ and CD276^high^ CSCs in the invasive tumor front, supporting that this phenotype of CSC in invasive niches might be controlled by molecular mechanisms.

In summary, in head and neck cancer, NK and T cells respond to different stimuli provided by the CSCs and other stromal cells on the TME and usually have limited anti-tumoral activity due to immunosuppression. The absence of the normal immune response on the TME is mainly due to the lack of activated CD8^+^ T cells and low expression of ligands and cytokines needed for NK cell activation, cytotoxicity, and expansion. These findings highlight the relevance of multimodality therapies that disrupt the TME to release NK and T cells in head and neck cancer from an unfavorable immune condition. As the CSCs are relevant actors in tumor development and progression and show immune privileges in the TME, combining different therapy approaches targeting CSCs and immunotherapy may contribute to achieving better clinical results in head and neck cancer patients.

## Author contributions

FX, JS, CR, and MR contributed to the conception and design of the study. FX and JS organized the database. FX wrote the first draft of the manuscript. JS, CR, and MR wrote sections of the manuscript. All authors contributed to manuscript revision, read, and approved the submitted version.

## Conflict of interest

The authors declare that the research was conducted in the absence of any commercial or financial relationships that could be construed as a potential conflict of interest.

## Publisher's note

All claims expressed in this article are solely those of the authors and do not necessarily represent those of their affiliated organizations, or those of the publisher, the editors and the reviewers. Any product that may be evaluated in this article, or claim that may be made by its manufacturer, is not guaranteed or endorsed by the publisher.
